# Sense-Making of Loneliness and Exclusion From Social Relations Among Older Adults in Sweden

**DOI:** 10.1093/geront/gnad005

**Published:** 2023-02-08

**Authors:** Axel Ågren, George Pavlidis

**Affiliations:** Department of Culture and Society, Linköping University, Campus Norrköping, Sweden; Department of Culture and Society, Linköping University, Campus Norrköping, Sweden

**Keywords:** Loneliness, Exclusion from social relations, Self-identity, Othering

## Abstract

**Background and Objectives:**

Loneliness and exclusion from social relations (ESR) are frequently addressed as public health issues for older adults. Public discourses potentially influence how loneliness and ESR are understood in society and experienced by the individual. The aim of this study was to analyze how older adults in different parts of Sweden use the discourses and concepts available to them to describe experiences of ESR and loneliness, and how these descriptions are used to construct a self-identity.

**Research Design and Methods:**

Qualitative semi-structured interviews were conducted with 30 individuals (14 men, 16 women) aged 67–87 years and living in Sweden. Emphasis was, in line with perspectives of discursive psychology, on how individuals draw on discourses to make sense of experiences. The empirical material was analyzed through an inductive process where we were open to finding concepts and themes.

**Results:**

Most participants emphasized the importance of not being lonely, considered achievable through maintaining an active lifestyle. “Othering” was taking place, where a general image of a “lonely” older adult was referred to when speaking about “others” loneliness. Those who expressed feelings of loneliness related these feelings to loss, being omitted, and other difficult life circumstances.

**Discussion and Implications:**

States of ESR were discussed more comfortably than loneliness, whereas various linguistic resources were used to distance themselves from loneliness. These findings indicate the need for further studies elaborating on how older adults make sense of ESR and loneliness and what implications this has for older adults’ well-being and identity making.

There is a major public attention and stereotypical portraiture of loneliness and states of exclusion from social relations (ESR) as predominately aging issues (Dykstra, 2009; Tornstam, 2007). Internationally, loneliness is addressed by aging policies as a societal challenge and a major public health issue that threatens the cohesion of aging societies (World Health Organization, 2021). In Sweden, loneliness in older age first gained public attention during the 1950s, through critical reports about the state of eldercare. A main argument in these reports was that eldercare institutions were characterized by environments that lacked stimulation, thus leading to extensive loneliness (Wersäll, 2006). Over the past decades, problems related to loneliness in older age have been increasingly addressed within the public sphere in Sweden (Ågren & Cedersund, 2020), propelled by reports that showcase the proportion of older persons living alone in Sweden to remain steadily among the highest in the world (Reher & Requena, 2018). Yet, studies on loneliness have placed Sweden as one of the least affected countries (Surkalim et al., 2022), a trend that holds true for the older population as well (Dahlberg et al., 2018; Sundström et al., 2009).

There are, however, both conceptual and cultural challenges as loneliness and ESR are often used interchangeably (Stein & Tuval-Mashiach, 2015), whereas relevant surveys often draw on similar factors and indicators for both concepts (Levasseur et al., 2010). This ambiguity has led scholars to challenge the current state of knowledge on the issues of loneliness and ESR in older age (Perissinotto & Convinsky, 2014), as the less-than-clear dissociation of the two concepts has important implications for clinicians and policy-makers (Valtorta & Hanratty, 2012). For example, older adults that are challenged by different conjunctions of loneliness and ESR states (i.e., lonely and excluded; lonely but not excluded; not lonely but excluded), would not benefit from the exact same intervention (Newall & Menec, 2019; Pavlidis, Motel-Klingebiel, & Aartsen, 2022).

A commonly used definition of loneliness is, within science, that of a *subjective feeling* of having fewer social relations than desired, or that of an unfulfilled intimacy from established social relations (de Jong Gierveld & Tesch-Römer, 2012; Perlman & Peplau, 1981). The *objective shortage* in one or more aspects that typify the structure of social networks (e.g., network size, meaningful relations) are defined as ESR states (Aartsen et al., 2021; Pavlidis, Hansen, et al., 2022). In that context, loneliness is often discussed as a potential consequence of ESR in older age. Loneliness and ESR states are, however, only weakly correlated in quantitative studies (Newmyer et al., 2021), which has been explained by the thesis that an excluded older person may not feel lonely and vice versa (de Jong Gierveld et al., 2018). Related efforts to examine, theorize, and discuss the conjoint conditions of loneliness and ESR in older age have been limited (Burholt et al., 2020; Dahlberg et al., 2022; Newall & Menec, 2019; Pavlidis, Motel-Klingebiel, & Aartsen, 2022). In addition, the reduction of both issues into the single and easily “digestible” message of the “loneliness epidemic” may contribute to homogenizing the objective lack of social relations with the subjective experience of loneliness (Snell, 2017), rendering both indistinguishable aging issues (Victor, 2015, p. 189).

The transformative changes in how information and knowledge about health, which we argue also applies to loneliness and ESR states, are produced and distributed in the media, political debates, policies, and science, have societal and cultural implications (Hallin et al., 2013). In line with these claims, Wood (1986) argues that loneliness is a private and simultaneously a social experience, because societal notions of social interaction affect how individuals understand what is missing in their social life. Rosendale (2007) argues that loneliness is often understood as a pathological state and social or personal deficit in need of correction, where the diagnostic process, in times of neuroscientific and psychopharmacological advancements, deconstructs suffering into a solvable problem. In late modernity, this may be rooted in the social imaginary of older age, namely the ways people imagine and evaluate their social existence shaping normative notions and expectations (Gilleard & Higgs, 2013).

Gilleard and Higgs (2013) argue that the social imaginary of older age is characterized by the medicalization of older age and its division between the third and fourth ages. On the one hand, the third age is a “cultural field” consisting of consumerism, cultural engagement, leisure, and engagement in technologies for self-care. On the other hand, the fourth age “can be better understood as representative of a feared ‘state of becoming’, an ascribed community of otherness, set apart from the everyday experiences and practices of later life. Its epigenetic ‘otherness’ is reflected through its representation within third person narratives by themes of abjection, frailty and marginalization” (Gilleard & Higgs, 2013, p. 368). These processes of othering were recently found in the context of retirement communities in the United Kingdom and Australia, where “them” was described as dependent and “us” as independent (Carr & Fang, 2022).

This social imaginary of older age may be reflected on discursive resources that older persons use to undergird a socially desirable self-identity that is closer to the third age (Alacovska & Kärreman, 2022). Furthermore, how older adults make sense of loneliness and ESR states are important topics of examination, given that their stereotypical descriptions may be internalized by older adults and used to construct a self-identity (Pikhartova et al., 2016). In line with overarching perspectives of discursive psychology, the language used is context bound and the words used give meaning to our experiences (Jørgensen & Phillips, 2002). Older adults may access discourses on loneliness and ESR states through interactions with others, or their exposure to the media, artistic expressions, and policies. There are several societal notions of aging, loneliness, and ESR states that older adults may draw on when making sense of individual experiences and constructing a self-identity along these concepts (Giddens, 1991; Wetherell & Maybin, 1996). Conducting in-depth interviews focusing on discourses embedded in older adults’ sense-making of ESR states and loneliness may highlight how older adults recount these issues to themselves when constructing a self-identity.

Because older persons have a significantly accumulated experience over the life course, it is of relevance to examine the ways in which older adults draw on life-course perspectives when constructing a self-identity and making sense of loneliness and ESR states (Holstein & Gubrium, 2007). The onset of older age is culturally and dynamically discussed, but only conventionally and arbitrarily set (in developed countries) at the age of 65 (Kowal & Dowd, 2001). Accordingly, the aim of this study is to examine how persons older than 65 in Sweden use discourses and concepts available to them to describe experiences of ESR states and loneliness today and over the life course, and how these descriptions are used to construct a self-identity.

## Method

### Design

When collecting and analyzing the data, we were guided by the key point of discursive psychology, which is that individuals draw on available discourses when shaping and presenting identities, emotions, and thoughts (Jørgensen & Phillips, 2002). Thus, “the self” is seen as constructed through ongoing interaction with the surrounding world, where cultural narratives and discourses position individuals in certain categories (Gergen, 1994), for example, as “older” or” lonely.” Qualitative interviews emphasize descriptions of lived experiences, perspectives, and accounts made by study participants (Kvale & Brinkmann, 2009). Prior to conducting the interviews for this study, an interview guide was developed with focus on social relations and loneliness in the past and the present. The interview guide was semi-structured with open questions, where we allowed space for the participants to put emphasis on questions of most importance to them. Examples of questions asked were: *Tell me a little about your daily routine and who you get to interact with on the daily basis; Who do you trust to discuss sensitive issues with; Have you ever felt lonely*? These questions did not address the issues of ESR states directly, yet they provided the grounds to gain insights into participants’ everyday life and social relations, which thereafter led to discussing ESR states. The questions included in the interview guide are attached in the Supplementary Section 1.

#### Recruitment

As part of a larger European research project that examined the potential gendered pathways of exclusion from social relations in older age, participants were recruited through an open call for participation, advertised to Swedish pensioner organizations. The call for participation did not make any reference to loneliness or ESR, rather than that called for participants that “would like to participate in study of social relations after retirement.” The call addressed persons 65 years or older living in Sweden, who do not live in care institutions or care settings, who are proficient in Swedish, and who have not been diagnosed with any form of dementia or any other severe neuropsychological disorder (e.g., schizophrenia, stroke).

The call for participation consisted of information about the study, how the interviews would be conducted, and the conditions for participating. We made clear that participants would remain anonymous and had the right to withdraw from the study at any time, without giving a reason for this. Persons interested in participating in the study contacted a research administrator and were given an information sheet and an informed consent to sign. All participants contacting the research administrator met the inclusion criteria of this study and thus were included in the sample. The recruitment began in February 2021 and was completed in April of the same year, approximately 12 months after the social distancing measures for those older than 70 were strongly recommended by the Swedish government (Ludvigsson, 2020). The recruitment ended based on meaning saturation, agreed by both authors, when data from interviews no longer contributed to new insights into the identified categories (Hennink et al., 2017). The study received ethical approval from the Swedish Ethical Review Authority (No. 2020-02045).

#### Participants

In total, 30 persons participated in the study, with a nearly equal gender distribution (14 men and 16 women). The participants were of postretirement age (*M* = 73.80, *SD* = 4.54, range = 67–87), and self-rated their health as very good or excellent (61.5%), good or fairly good (30.8%), or less than good (7.7%). From the participants, 28% reported mobility restrictions due to health issues. Most of the participants were living with their spouse (73%), some were widowed (11.5%), divorced (11.5%), and one was never married. Most of the participants had an equivalent to high school education (73%), with 11.5% having more and 15.4% having less education.

### Data Collection

The interviews lasted from 37 to 85 minutes and were conducted over the phone, due to ongoing restrictions during the Covid-19 pandemic, by one of the authors and a research assistant. Throughout the interview, the interviewer was attentive to what topics participants were most interested in discussing, which guided the direction of the interviews. Thus, the semi-structured interview protocol allowed study participants to reflect upon and discuss topics addressed during the interview and focus in-depth on issues that were most important to them. At the end of the interview, the participants filled out a short demographics and self-reported health questionnaire. The questions included in the questionnaire are attached in the Supplementary Section 2.

### Data Analysis

Each interview was audio-recorded and subsequently transcribed verbatim and analyzed in their original language (i.e., Swedish), with the excerpts included in this article translated from Swedish into English by the authors. When analyzing the empirical material, we used the general inductive approach for qualitative data (Thomas, 2006), where we were open to finding new concepts and themes in the data analyzed. Moreover, the process of coding was inspired by constructivist grounded theory as developed by Charmaz (2006), with emphasis on how participants construct meaning in certain situations and contexts, and how individuals make sense of own experiences through language use in certain contexts (Jørgensen & Phillips, 2002). Both authors were involved in coding and analyzing the transcripts. The first author had already experience in coding and analyzing qualitative data with these methods, whereas the second author was novel in this process. The authors met regularly and at least once after each coding phase to compare and discuss findings.

The process of coding started with first familiarizing ourselves with the material through reading and re-reading the transcribed interviews (Phase 1), noting on the overall feeling of what the participants were saying. Thereafter, we proceeded to initial coding (Phase 2), where the focus was on words, concepts, and phrases which highlighted, in line with the aim of the study, how participants made sense of ESR and loneliness, as well as how these descriptions were used to construct a self-identity. As we adopted an inductive approach, the focus in this phase was on identifying meaning units within the data and labeling and categorizing them into codes (Thomas, 2006). For example, one participant spoke about how he/she was not lonely because he/she was an “outgoing person,” summarized by the codes “Outgoing person” and “Not lonely.” During Phase 2, we found that ESR states were described in detail using phrases like “I do not see anyone” or “I come home, and I am alone,” and not with the use of scholarly phrases like “I am excluded.” These phrases were regarded as meaning units that conveyed issues under the code “ESR states.”

**Figure 1.**
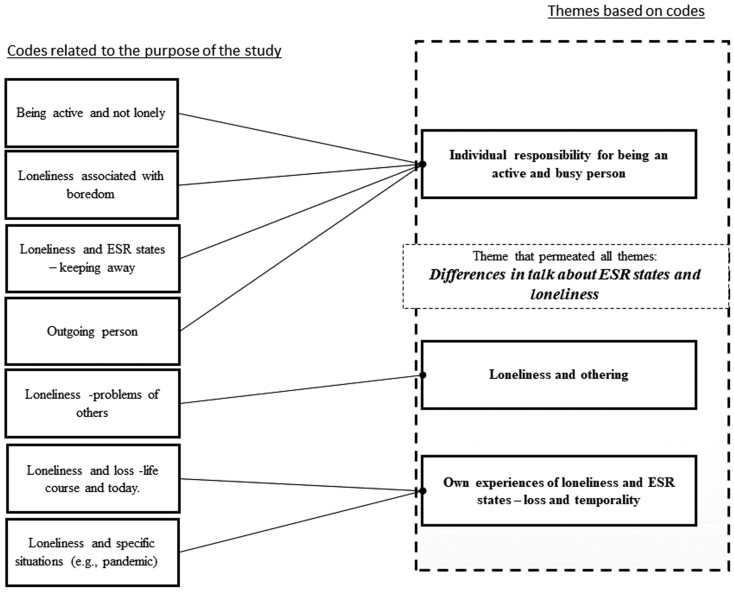
Examples of themes stemming from codes that highlight how the participants made sense of loneliness and states of exclusion from social relations (ESR).

In the final phase, Phase 3, we proceeded to what Charmaz (2006) calls focused coding, where the task is to identify significant categories, based on codes found, and thus decide which codes are most important to proceed within the analysis. This phase was the crucial step from codes to themes, a process that is not straightforward. Concurring with Hennink et al. (2017), the transition from codes to themes took place when the material identified was rich and contributed to an in-depth understanding of the concepts and expressions used by the participants. Examples of themes stemming from codes can be found in Supplementary Section 3 and Figure 1.

## Findings

In this section, we will present the themes found after analyzing the empirical material from the 30 interviews conducted in this study. The excerpts corresponding to the excerpt’s codes are listed in Table 1.

**Table 1.** Excerpts Codes and Excerpts of Older Persons’ Accounts on Loneliness and Exclusion From Social Relations

Excerpt code	Excerpts
E1	“…it is a depopulated village, not many people live here [...] and there are several empty houses there and it is very sad that no people live there. […] So on a normal day, I do not see anyone, I may not see any neighbours at all. Especially not during the winter, because then you are not outdoors.”
E2	“… the last three weeks have actually been quite interesting because then I felt like the first day, then I called my friends and cried and was like just blistered and all. But then I felt like this, now many people have asked me (how I feel) ‘Yes, but I enjoy being alone’, I felt great. I married late and lived alone for a long time and didn’t feel bad about it either, there are so many other things I enjoy doing.”
E3	“I can say the opposite (to feeling and being lonely), that I have felt terrified of being alone. But I’ve made sure I’ve had these contacts. And as I said, I have been involved in associations from the beginning to the end here.”
E4	“I’m trying to keep myself pretty busy. […] since then, I have probably never had those problems (referring to experiencing loneliness), because I have had interests that I am working on”
E5	“If you do not want to be alone, then you do not need to be. [..] if you have not hung out or had so many around you then. And suddenly you are alone.”
E6	“‘Now I feel bored’, if you have those thoughts: ‘What should I do now?’ And then it’s a matter of maybe calling someone, or you must be active, you know you’ll come up with solutions for that. […] If someone has moved away or something like that: ‘Oh, now I must deal with this!’ It feels like you must be strong not to end up in loneliness.”
E7	“…if I think I’m a little bored then I get in touch with someone and then we do something.”
E8	“…my neighbour, those who did not have any children, they did everything together, she and her husband. And then a few years ago we were out eating New Year’s lunch, […] And then he died suddenly when we were out eating. […] And she’s really lonely, although she never complains about loneliness or anything, because she also has many interests. And then we talk to each other every day and either I call or she calls me and just asks how things are going and so on or also if I have not heard anything and that, I check if she has taken in her newspaper.”
E9	“She (a former colleague) came with a walker and she had broken one leg and so she talked about her days, totally alone, with children far away in the country and (she) sat alone.”
E10	“I’m an only child, I have no siblings. And then I could, when my father died earlier and my mother died just over ten years ago and such situations, even though I then had children and was married of course, you feel lonely and abandoned. Especially when my mother died because she was the last (oldest person in her family), then I was the oldest and alone in that way, and alone in a way even though I had a family.”
E11	“… during the funeral itself and at this moment of remembrance afterwards, it was full of people and relatives and friends and that was when you could still meet at a funeral (referring to restriction measures against the spread of the coronavirus). And then, it went well, like, when you had people around you and everyone came and comforted you and so on. And then, after that I was driven home by my daughter, and she has her own family, so she just dropped me off here where I live, and then she went home. And it’s my own fault, I feel like I could have said to her: ‘Please, can you be with me tonight, because I feel so lonely and sad!’ I did not, because she was also very sad, and she needed to get home to her children and so on. But that night, I know it was awful. […] I had been alone for many years then, so that I, as it was, I was used to loneliness, but just at that moment I missed a shoulder to lean on, if you like.”
E12	“So, I can also feel lonely sometimes and yes, I know on bigger holidays and so on, of course I meet my children and so on, but in the end you come home to the loneliness on Christmas Eve and then it can also feel a little lonely when you know that, that others are sitting together.”

### Differences in Talk About ESR States and Loneliness

The differences in how ESR and loneliness were described permeated all themes. One example of how participants made sense of ESR states was found in SE_210208_01_EE’s account (see E1 in Table 1). The participant emphasized how the physical environment contributes to ESR states. When discussing the specific issue of loneliness, however, the participant stated that experiences of loneliness mainly occurred in brief periods earlier in life and nowadays were associated with not being able to meet others due to the coronavirus pandemic. Not meeting others was an ongoing issue during the time of the study’s interviews, although the recommendations by the Swedish government for persons 70 years and older not to meet others outside the household were not legally binding (Ludvigsson, 2020). In general, the participants were very keen to describe social relations, such as their upbringing, their relations with family members and friends, as well as the social aspects of everyday life. As illustrated in excerpt E1, participants could talk about ESR states and adversities extensively and in detail. When it came to experiences of loneliness, however, this was not elaborated on and not seen as a problem, or if so, only to a minor extent. The perhaps noteworthy issue here is that for the participants who focused on negative life events, such as loss and solitary living situations, loneliness was not presented as persistent nor expressed as a problem.

One example of this is highlighted by SE_210211_01_AÅ, whose husband recently developed dementia (see E2 in Table 1). First, she described in detail the adversities faced when her husband developed dementia and how she eventually “hit the wall” and how upset she was when her husband moved to a nursing home. Her experience of her husband eventually getting an apartment in a nursing home was described thereafter in terms of elated ESR. This did not, however, lead to feelings of loneliness as she enjoyed being alone and “felt great.” Moreover, loneliness was seen through a life-course perspective where being used to living alone earlier in life and having “many other things” resulted in her not seeing loneliness as a problem.

### Individual Responsibility for Being an Active and Busy Person

Several interviewees stressed the importance of having interests, hobbies, and being active. The significance ascribed to being active was not articulated within a specific rationale of reducing loneliness, because the participants, as previously mentioned, only talked about experiencing loneliness in some few cases. Rather than that, presenting oneself as active was clearly described as the means to keep ESR states and loneliness away, as illustrated in excerpt E3 (see Table 1) from SE_210210_01_EE. There, fear of loneliness and ESR are illustrated as a motive for being active in associations and maintaining social contacts.

Presenting oneself as an active person was often addressed through a life-course perspective, where participants talked about how they have been active in associations throughout their lives and how this lifestyle has been upheld later in life. For SE_210210_02_AÅ (excerpt E4, see Table 1), loneliness was understood as being kept away through a busy lifestyle, involving hobbies, interests, upholding social relations, and participating in associations. Looking at these accounts through the lens of discursive psychology and how people construct a self-identity (Wetherell & Maybin, 1996), the use of the phrase “busy” and its associated concepts is a type of linguistic resource used to describe how the participants are not lonely and not excluded from social relations. Thus, to describe themselves as non-lonely means to construct a self-identity of a busy and active person.

As highlighted earlier, loneliness was avoided as an explicit account among the interviewees; instead, and similar to the findings by McHugh Power et al. (2017), “boredom” or the “fear of being alone” was mentioned as resulting from not meeting others, not participating in activities, not getting involved in associations, or not being able to work on hobbies. Underlying this understanding was the notion of agency (MacLeod et al., 2019) and individual responsibility, as participants recurrently stressed how it is up to oneself to find ways to avoid ESR states—as found in the reflections made by SE_210215_02_EE, when the discussion about loneliness became more general (excerpt E5 in Table 1). Similar to other participants, SE_210215_02_EE’s account can be seen through a constructionist perspective on the life course (Holstein & Gubrium, 2007), where ESR states made sense by linguistically drawing on consequences of choices made earlier in life and their influence on the current life circumstances in older age.

Along the same lines, SE_210217_01_EE used loneliness and boredom interchangeably to describe negative feelings, the latter resulting from earlier choices and actions in life, where the notion of individual responsibility and agency is clearly described in the participant’s account (excerpt E6, see Table 1). It is worth noting the general focus in SE_210217_01_EE’s account, where references to loneliness (or boredom specifically) are not made to his own life, but a general description of how a person should act when feeling “bored.” Furthermore, this description is of interest as he describes ways of alleviating “boredom” and explicitly stated loneliness, where being active and social is a matter of simply coming up with solutions and calling someone. Thus, from this perspective, it is a matter of individual responsibility, where the individual can choose from various strategies for not being “bored” or lonely. Simultaneously, however, he argues that you need to be “strong not to end up in loneliness,” which indicates that these efforts are not so easily carried out.

SE_210301_01_AÅ presented a similar view on this issue but, in contrast to the other participants, she explicitly referred to her own life as found in this excerpt (excerpt E7, see Table 1). As shown, SE_210301_01_AÅ presents a somewhat rational description when feeling “bored,” where it is simply a matter of calling someone and thereafter doing “something” together. Moreover, SE_210301_01_AÅ highlights how this view is part of constructing a self-identity, when presenting herself as a capable actor who can “get in touch” with someone when feeling bored. Overall, the recurring perspective was that alleviating loneliness, or “boredom” as loneliness was more comfortably referred to, was subject to individual responsibility. This indicates that the participants took the view that persons who are capable of choosing between various actions (Jolanki, 2015) can alleviate the burden of loneliness and ESR states.

### Loneliness and Othering

Concurring with the argument of Jensen (2011) in the Nordic context, othering in our study took the form of the “self” being positioned as the other, and by refusing to occupy the position of the other by disidentification and claims to normality. As the account of SE_210217_01_EE illustrated earlier (i.e., “you must be strong not to end up in loneliness”), loneliness was described more comfortably by using expressions in a general sense, or when other people who are not able to manage loneliness were used as examples.

Yet, othering served also to mark those who are different from the “self” (Weis, 1995). An example of this was found in the interview with SE_210215_02_EE, who talked about a “lonely” neighbor (excerpt E8, see Table 1). This displays several aspects of ESR states and loneliness but implies an othering of loneliness. The construction of the neighbor as a “lonely person” in the SE_210215_02_EE account implies an ESR state with a spatial element, as the neighbor was described as living alone. But at the same time, this excerpt contains a contradiction, as the neighbor was described on the one hand “very lonely,” but on the other hand as having daily contact with SE_210215_02_EE and not complaining about loneliness.

This raises the question of how excluded (from social relations) that neighbor really was, and how the process of constructing others as “lonely” may be based on certain cultural understandings of ESR and loneliness. Here, loneliness is equated with living alone, having experienced loss, and not having a spouse or family members, which resonates more closely with scholarly definitions of ESR states. However, when SE_210215_02_EE returned to her own life, she described how she was “left alone” when her husband died and that she had no relatives. Despite this description of being “left alone,” she never referred to herself as a “lonely” person or having persistent feelings of loneliness, suggesting that loneliness is more easily used to construct identities for others than for oneself.

Another example of othering came in the interview with SE_210202_01_EE (excerpt E9, see Table 1). For SE_210202_01_EE, a specific ESR state was associated with poor physical health and reduced mobility, giving a spatial element to the notion of being “totally alone,” referring to geographical remoteness from family members. However, the process of othering in the accounts of SE_210202_01_EE lies in the descriptions of his own experiences of loneliness, which was not caused by objective ESR states. Loneliness, he argued, is a matter of how it is interpreted and that *you* can feel lonely in the mind even if *you* are not physically alone. Consequently, he distanced himself from loneliness by talking about “you” in a general sense, hence without using “I” as a pronoun that implies “the self” as an active agent in his own story. Later in the interview, SE_210202_01_EE, related feelings of loneliness to his marital life, which over 50 years of marriage had ups and downs, and it has been “hell” on several occasions. The couple never discussed the possibility of divorce, and now they are, according to him, “too old” to be upset with each other. The descriptions by SE_210202_01_EE were involving processes of othering, talking about loneliness in general terms, but simultaneously also expressing feelings of loneliness as related to his own marital challenges.

### Own Experiences of Loneliness and ESR States—Loss and Temporality

In several interviews, loneliness was described as a temporal feeling with a beginning and an end, whereas ESR was described as a more persistent and often irreversible state, especially when linked to loss. An example comes from the account of SE_210218_01_EE (excerpt E10, see Table 1). What appears here are descriptions of loss that do not lead to an ESR state (i.e., she was married and had children), but do cause feelings of loneliness. Here again, accounts of loneliness consisted of talking about “you” in a general sense, and not the pronoun “I” that implies “the self” as an owner of the feelings of loneliness and abandonment.

Further on in the interview, SE_210218_01_EE gives an account of temporality in her feelings of loneliness, stating that she did not feel lonely at present. Nevertheless, “coronavirus thoughts” and the fact that her husband still worked resulted in her being home alone during the day, contributing to feelings of being “omitted.” With that reference, she portrayed her upbringing as less “lonely,” because she lived with her parents, uncle, aunt, and cousins when she was growing up. Moreover, according to this participant, family ties were stronger in the “old days,” as people lived more together, across generations, and closer to each other. In parallel, she drew on the irreversible nature of ESR states “nowadays,” citing a contemporary societal trend of increased mobility where daily contacts “cease to exist.”

This illustrates how, using certain discourses and concepts, she intertwined personal experiences with larger societal developments when making sense of her own life, and experiences of loneliness and ESR states, although stating that “you” and not “I” “feel lonely and abandoned.” These ways of relating loneliness to a critique of the development of “modern” society relate to findings by Schirmer and Michailakis (2015), where individualization and loss of smaller communities are used as symbols of increased loneliness in contemporary society.

The descriptions given by SE_210211_02_EE stand out in comparison with how the other participants talked about loneliness. For her, loneliness was a highly present state and experience which had several facets. Loneliness was related both to everyday life and specific occasions throughout life, giving as an example her mother’s funeral (excerpt E11, see Table 1). What appears in this excerpt is, in line with what Graneheim and Lundman (2010) also found, how loneliness is linked with loss and described as an intense experience, here constrained timewise to a specific night and situation. But later in the interview, SE_210211_02_EE referred to several situations in her everyday life where feelings of loneliness were present with a start and an end. One example was when she watched TV programs and was not able to discuss the content with others. Another example was the festive holidays (excerpt E12, see Table 1).

Here we see another “othering” of loneliness, where she comes home to “the loneliness,” whilst others are “sitting together.” Thus, she referred to recurring events that reflect ESR states, leading to feelings of loneliness, such as being home alone on holidays, combined with a notion of others being in the company of each other and less lonely than herself. Consequently, ESR states leading to feelings of loneliness merged with a type of “othering,” serving to reinforce her self-identity of being an excluded and lonely person.

## Discussion

The aim of this study was to examine discourses and concepts used to describe experiences of loneliness and ESR, and how these descriptions are used in the process of constructing a self-identity among older adults. We have been guided by discursive psychology’s view on language as context-bound representations of the individual social world, implying that experiences and identities have no predetermined inner “essence” (Jørgensen & Phillips, 2002). Instead, using discourses rooted in the social imaginary of older age, older adults contribute to the production of, and give meaning to, their experiences of ESR states and loneliness, relating them to self-identities or the identities of others.

Although the themes found display variations and similarities within and between them, we found that all derive from a larger discourse, namely that of being (or not being) active. This overarching understanding permeated the articulation of perspectives on ESR states and loneliness, where being active in a broad sense was the positive and desirable goal. This dominating perspective served, in turn, to construct loneliness as persistently associated with what were understood as negative characteristics, such as fear of being alone, boredom, illness, ESR states, and loss. Thus, the participants reflected on common cultural distinctions between the “third age,” the period of activity and opportunities, and the “fourth age” as the new “real old age” associated with inactivity, frailty, illness, loneliness, and death (Higgs & Gilleard, 2014; Reul et al., 2022).

Although this may contribute to “the chimera of younger adults’ health in an older adults’ body” (Martin et al., 2015, p. 22), the participants drew on illness, frailty, and being alone to describe how others were lonely, whereas overall presenting themselves as active and being “too busy” to feel lonely. Thus, the fears of loneliness were embedded in fears of what constitutes a social imaginary of the “fourth age” (Gilleard & Higgs, 2013), namely a period characterized by passivity, decline, ESR states, and dependency. Meanwhile, the ways of talking about avoidance of loneliness entailed a process of “othering.” More precisely, the ways of describing loneliness were part of a process of constructing the identity of others as lonely, even when seemingly they were referring to others’ ESR states (e.g., friends or neighbors living alone) and not their feelings of loneliness per se. This type of othering resembles findings by Carr and Fang (2022), where “them” was described as dependent and/or facing deteriorating capabilities, whereas “us” was described as independent and active.

It is noteworthy that ESR states were discussed more comfortably in comparison with loneliness. Participants described various adversities over the life course and in later life, such as loss, difficulties during childhood, conflicts with children, diseases, spouse developing dementia, problems during working life, and living in depopulated villages. However, very few of the participants described their lives and identity in terms of loneliness. These findings raise the question of how to understand the linguistic resources used when discussing individual experiences of loneliness and ESR states in older age, and how to distinguish between them in older adults’ accounts of these experiences. We argue that these findings are in line with findings from Róin et al. (2021), where octogenarians used various terms to present positive images of themselves, focusing on downplaying health problems through humor, comparing themselves with others to present themselves as more active and younger than their chronological age (Róin et al., 2021).

Victor et al. (2009) claimed that respondents do not want to admit to feelings of loneliness in studies and surveys as it may compromise their experienced self-worth, leading to answers that are publicly acceptable and ego-defensive (Solano, 1980). Admitting to loneliness may be embarrassing and associated with failure, passivity, and stigma (Griffin, 2010; Rokach, 2013; Shiovitz-Ezra & Ayalon, 2012). Thus, not admitting to loneliness could be understood in terms of not wanting to break the happiness barrier, which is argued to be a strategy and subjective mental structure where unhappiness, despite difficulties, is downplayed by oneself (Roos, 1988).

We could not, however, know whether the participants did not want to admit to feelings of loneliness during the interviews or whether the reluctance to describe experiences of loneliness is rooted in broader societal understandings of loneliness as being surrounded by stigma (Rokach, 2013). Constituting a limitation of this study, we did not actively seek to recruit older persons that identify themselves as excluded or lonely, thus may have not captured the social construction of these concepts among those who admittedly suffer the most by these states and feelings. Despite that, talking about ESR states, even when it was resulting from loss, living alone, depopulation, and having faced various adversities throughout life or in specific situations, was seemingly not an equally difficult topic to be addressed by the participants of this study as loneliness was.

What appeared in the interviews was that various linguistic resources and strategies were used to downplay experiences of loneliness, by distancing oneself, talking about loneliness in general terms and depicting loneliness as a problem of “others,” whereas the accounts of ESR states were more explicit referred to the “self.” Consequently, in line with arguments by Jylhä and Jokela (1990), we argue that there is a reluctance in defining oneself as “lonely” in terms of subjective feelings of loneliness, but not equally so in terms of describing ESR states. It is, however, of importance to emphasize that two participants, an important minority of the sample, described themselves as lonely and provided detailed descriptions of situations leading to loneliness. For these two persons, loneliness was described as the results of loss and not sharing everyday life with someone else, hence a description of persistent ESR states.

The findings of this study contribute to insights on how older people in Sweden make sense of loneliness and ESR states and how not being lonely is important in constructing their self-identity aligned with cultural understandings of active and “busy” lifestyles (Ekerdt, 1986). We illustrated ways in which linguistic resources were used to make sense of loneliness through a process of “othering,” whereas ESR states were described more comfortably as a living condition of the “self.” These findings have implications for research on loneliness and ESR states, because older adults seemingly strive to downplay their own feelings of loneliness and avoid the identity of a lonely person. In times when interventions to reduce loneliness are frequently carried out, these findings have implications for practitioners who are called to identify lonely older persons who would benefit the most from such interventions. Nonetheless, the findings of this study highlight the need for further research on the ways older adults make sense of loneliness and ESR states, the conceptual dissociation of these two concepts, and how to support older persons who are challenged by conjoint types of relational shortages.

## Supplementary Material

gnad005_suppl_Supplementary_Material

## Data Availability

The data are available in their original form (transcriptions in Swedish) upon request to the Division of Ageing and Social Change, Department of Culture and Society, Linkoping University.
