# Supplementary material for: Sense-Making of Loneliness and Exclusion From Social Relations Among Older Adults in Sweden
Source: Gerontologist. 2023 Feb 8;63(7):1140–8. doi: 10.1093/geront/gnad005 (PMC10448986; doi:10.1093/geront/gnad005)
Supplement: gnad005_suppl_Supplementary_Material [file gnad005_suppl_supplementary_material.docx]

***Section 1: Interview Guide***

1. **First tell me a little about your daily routine and who you get to interact with on the daily basis?**

What are the opportunities available to you for meeting others? Where do you go to meet people and interact with them socially? For example, general day to day activities, clubs, societies?

- How was it changed during the recent Coronavirus pandemic? How did it make you feel? How did you cope with these changes?

1. **Looking across your network: how has it changed across your life course?**

Can you tell me who you feel the closest to? If you feel close to more than one person, that is ok, which person(s) do you feel closest to?

Who do you trust to discuss sensitive issues with?

Looking across your network how would you describe the quality of relationships across your network?

Looking back at your life, have you ever lost or ended a relationship with people that were close to you? Can you tell me a little about these experiences? Can you please add the names and types of relationship you had with them in parentheses?

1. **Do you think that being a men/woman has influenced the relationships you have?**

Growing up, do you think there was an expectation around how people socialized and the kinds of relationships they should have, such as for men and women?

1. **Social relationships can also be difficult and distressing at times. Have you ever experienced anything like that? (for example, distress, conflict, or any type of type of mistreatment). Please tell me a little bit about this.**

Given your experience of this/these difficult relationship(s), do you think they are generally the same for men and women?

1. **We talked about people who are important in your life and conflicts you experienced. Now I would like to ask you about those moments when you feel somewhat lonely. Have you ever felt lonely? Can you tell me about these experiences?**

Has your feeling of loneliness changed across your life course? (has it always been like that?)

Would you say feeling lonely impacted on you in any ways?

Do you think this has to do with the way men and women build relationships differently?

How was it during the recent Coronavirus pandemic? How did it make you feel? How did you cope with these changes? Do you think it was the same for women and men?

1. **Have you ever experienced a situation where you would have needed support and didn’t receive it from the people we talked about earlier/those in your social network? Can you tell me a little bit about these experiences?**
2. **On the contrary: When you look back at your life, do you recall any situations in which someone significantly supported you in dealing with problems or challenges? Can you tell me a little bit about these situations?**

How was it during the recent Coronavirus pandemic? How did it make you feel? How did you cope with these changes

Do you think it was the same for women and men?

1. **We talked about different sorts of supports that you get from your social network. Now I would like to ask you about support you provide to people (in your community) and how this is related to community engagement and civic activities. In what ways, if any, have you ever provided support to others across your life course?**

Have you ever got involved in your local community across your life course?

Have you ever supported others by volunteering across your life course?

Have you ever participated in political activities, such as working on political campaigns, signing petitions or participating in protest activities, across your life course?

1. **We talked about different sorts of supports that you provide and ways you engage in social activities. Now I would like to ask you if there is anything that prevents you from participating more in social relationships or social activities? If so, what?**

Do you feel your age prevents you from participating more in social relationships or social activities?

Do you feel your gender prevents you from participating more in social relationships or social activities? Has it always been like that?

Do you feel your financial situation prevents you from participating more in social relationships or social activities? Has it always been like that?

Do you feel your health prevents you from participating more in social relationships or social activities? Has it always been like that?

1. **Now we have talked about barriers to social participation you have faced or are currently facing. Now thinking about the future, is there anything you would like to change about your social relationships that might make your life better?**

What would you need to change it?

1. **Coming to the end of the interview I would like to ask: Reflecting back on your life, what have social relationships meant to you and what have they contributed to your life?**

What have they contributed to your health?

What have they contributed to who you have become (are now)?

What have they contributed to the way you dealt with the Coronavirus pandemic?

1. **Is there anything you would like to add?**

Were there any questions in the interview that were complicated for you or felt somewhat irritating?

***Section 2: Demographics and Self-Reported Health Questionnaire***

1. Age

In which year were you born?______________________________

In which month were you born? ______________________________

1. Gender:

- Male
- Female
- Other

1. Were you born in (COUNTRY of interview):

- Yes IF Yes, go to 8

No

1. In which country were you born? Please name the country that your birthplace belonged to at the time of your birth. ___________________
2. In which year did you come to live in (COUNTRY of interview)?___________________
3. In what year did you become a citizen of (COUNTRY of interview)?__________________ (did not become possible answer)
4. What is your marital status?

- Married and living together with spouse, (year of marriage________________)
- Registered partnership, (year of registering partnership_________________)
- Married, living separated from spouse, (year of start of separate living______)
- Never married
- Divorced, (year of divorce_________________)
- Widowed, (year of death of the spouse____________________)

1. Who is living with you in your household (multiple answers possible):

- Live alone
- Partner or spouse
- Children
- Others-___________________

1. What is the highest school leaving certificate or school degree that you have obtained?

- No education
- Public school (less than 6 years)
- Public school (6-8 years)
- Public school plus vocational training min. 1 year
- Public school and läroverk 8 years
- Primary and lower secondary education (9 years)
- High school
- High school plus min 1 year vocational education
- I am still studying
- Other type

1. What would you consider to have been/to be your primary occupation? Please give the exact name or title____________________________________
2. How would you describe the area where your residence is located?

- A big city
- The suburbs or outskirts of a big city
- A large town
- A small town
- A rural area or village
- A remote rural area

1. The level of monthly income for single 65+ person in [country of interview] is [enter value in local currency] gross. How would you describe your level of income?

- Much below average
- Some below average
- Like average
- Some above average
- A lot above average
- Prefer not to say

1. Thinking of your household's total monthly income, would you say that your household is able to make ends meet...?

- With great difficulty
- With some difficulty
- Fairly easily
- Easily
- Prefer not to say

1. In general, would you say your health now is…

- Excellent
- Very good
- Good
- Fair
- Poor

1. Are you limited in your ability to carry out normal everyday activities, because of a physical or mental health problem or a disability?

- Yes
- No
- Does not know
- Prefer not to say

***Section 3: Examples of Coding into Themes***

During the data analysis, we found that several participants highlighted the importance of being “active” and “having interests” as a strategy for “not being lonely”. Consequently, these statements and the respective codes were categorized in Phase 3 under the theme “Individual responsibility for being an active and busy person”.

Equally important, however, was *how* the participants talked about these certain issues, where we were open in findings variations within themes. The codes identified by us could vary in content, but remained still within the same theme, since participants, overall, used similar concepts to describe certain issues. Specifically for the *how* participants talked about issues, we were also open to how significant certain issues could be for the participants. One example of this was when two participants extensively talked about loss of family members and living alone and related this to loneliness. Thus, although only addressed by two participants, the significance ascribed to these issues, based on having found significant codes in these two interviews, led us to identify the theme “Own experiences of loneliness and ESR states – loss and temporality”*.*

In line with the overall focus of discursive psychology on positioning, we paid attention in Phase 3 to how study participants positioned themselves through shifts in pronouns from, for example, “I” to “you”. Through the focus on positioning, it was also possible to identify differences in how participants talked about ESR states and loneliness, where ESR states could be described in detail and related to oneself. Loneliness was instead avoided through different linguistic strategies, as participants talked about “you” feeling lonely, whereas when ESR states were addressed, they used the “I” pronoun. In this context, participants distanced themselves from loneliness by referring to, for example, others being lonely, or to someone who would feel lonely in the specific situation. This led us to find the theme “Differences in talk about ESR states and loneliness”, which also transcended all themes. Moreover, since the process of othering loneliness was indeed prominent, “Loneliness and othering” constituted a theme of its own.

Guided by perspectives of discursive psychology, it was possible to discern accounts made by the participants which intertwined individual experiences of loneliness and ESR with larger discourses and concepts. One example of this was when a participant talked about feeling less lonely during childhood, since families were closer in the “old days”, thus drawing on *a general notion of societal development*. Another significant example of this was how several participants, in different ways, stretched the importance of being active, which relates to *fears of decline and dependency*, which are highly present issues in society today.
